# A third dose SARS‑CoV‑2 BNT162b2 mRNA vaccine results in improved immune response in hemodialysis patients

**DOI:** 10.48101/ujms.v127.8959

**Published:** 2022-10-13

**Authors:** Jan Melin, Maria K Svensson, Bo Albinsson, Ola Winqvist, Karlis Pauksens

**Affiliations:** a Department of Medical Sciences, Renal Medicine, Uppsala University, Uppsala, Sweden; b Department of Medical Biochemistry and Microbiology, Zoonosis Science Centre, Uppsala University, Uppsala, Sweden; c Laboratory of Clinical Microbiology, Uppsala, Sweden; d Department of Clinical Immunology, Karolinska University Hospital, Stockholm, Sweden; e ABC Labs, Solna, Sweden; f Department of Medical Sciences, Infectious Medicine, Uppsala University, Uppsala, Sweden

**Keywords:** Hemodialysis, chronic kidney disease, humoral and cellular immune response, SARS-CoV-2 BNT162b2 mRNA vaccine

## Abstract

**Background:**

The hemodialysis (HD) population has been a vulnerable group during the coronavirus disease 2019 (COVID-19) pandemic. Advanced chronic kidney disease with uremia is associated with weaker immune response to infections and an attenuated response to vaccines. The aim of this study was to study the humoral and cellular response to the second and third doses of Severe Acute Respiratory Syndrome Coronavirus 2 (SARS‑CoV‑2) BNT162b2 mRNA vaccine in HD patients and to follow the response over time.

**Methods:**

The patients received their first two vaccine doses from 28 December 2020 within a 4-week interval and the third dose in September 2021 and were followed-up for humoral and cellular immune response at 1) 7–15 weeks and 2) 6–8 months after dose two (no t-cell reactivity measured), and 3) 3 weeks and 4) 3 months after dose three. Fifty patients were initially enrolled, and 40 patients were followed during the entire study. Levels of COVID-19 (SARS-CoV-2) IgG antibody against the Spike antigen (anti-S) and T-cell reactivity testing against the Spike protein using Enzyme-Linked ImmunoSpot (ELISPOT) technology were evaluated.

**Results:**

IgG antibodies to anti-S were detected in 35 (88%) of the 40 patients 7–15 weeks after vaccine dose two, 31 (78%) were positive, and 4 (10%) borderline. The median anti-S titer was 606 Abbott Units/milliliter (AU/mL) (interquartile range [IQR] 134–1,712). Three months after the third dose, anti-S was detected in 38 (95%) of 40 patients (*P* < 0.01 compared to after dose two), and the median anti-S titer was 9,910 AU/mL (IQR 2,325–26,975). Cellular reactivity was detected in 22 (55%), 34 (85%), and 28 (71%) of the 40 patients, and the median T-cell response was 9.5 (IQR 3.5–80), 51.5 (14.8–132), and 19.5 (8.8–54.2) units, respectively, for 6–8 months after dose two, 3 weeks, and 3 months after dose three.

**Conclusions:**

Our data show that a third dose of SARS‑CoV‑2 BNT162b2 mRNA vaccine gives a robust and improved immunological response in HD patients, but a few patients did not develop any anti-S response during the entire study, indicating the importance to monitor the vaccine response since those who do not respond could now be given monoclonal antibodies if they contract a COVID-19 infection or in the future antivirals.

## Introduction

The hemodialysis (HD) population has been a vulnerable group during the coronavirus disease 2019 (COVID-19) pandemic, and data from the Swedish Renal Registry show that during the period from March 16, 2020 to March 15, 2021, 3% of all dialysis patients died due to COVID-19 in Sweden (1). This is also in line with a British study that showed in-center HD patients have had a higher incidence of COVID-19 infection due to more frequent healthcare contacts, and when infected HD patients often become more severely ill resulting in a higher mortality (2).

Chronic kidney disease (CKD) with uremia is associated with weaker immune response to infections and an attenuated response to vaccines against Hepatitis-B and seasonal flu (3). B lymphocyte and CD4+ T lymphocyte are decreased in this population as well as the T-cell response to antigenic stimuli. Moreover, impaired monocyte functioning results in inadequate antigen presentation to the antigen-presenting cells, generating weakened memory cells and inadequate antibody production after vaccination. Patients with CKD are known to have impaired function of neutrophils, with a lower capacity of phagocytosis and a greater rate of apoptosis although their number remains unchanged (4). The underlying mechanisms of the impaired immune system in CKD are multifactorial. Besides, uremic toxins, oxidative stress, endothelial dysfunction, low-grade inflammation, as well as mineral and bone disorders are involved and may contribute to the impaired immune system in these patients (5). It is therefore not surprising that HD patients have a weaker immunological response to COVID-19 vaccines compared to the general population (6).

Late in 2020, vaccines against COVID-19 became available, and beginning in December 2020, vaccination with Severe Acute Respiratory Syndrome Coronavirus 2 (SARS‑CoV‑2) BNT162b2 mRNA vaccine started to the patients of the two dialysis units of Uppsala University Hospital. We have previously reported levels of antibodies and T-cell reactivity 7–15 weeks after two doses of SARS‑CoV‑2 BNT162b2 mRNA vaccine, and we found that the majority of the HD patients had a clear immune response to the vaccine, but approximately 20% had a limited response (7).

The humoral response rate to the SARS‑CoV‑2 BNT162b2 mRNA vaccine has been found to be 75–97% (8), but the serological effect of two doses has been shown to decrease with time in both the general population (9) and in HD patients (10). Several recent studies have shown that a third dose of the BNT162b2 mRNA vaccine booster has immune response also against the SARS-CoV-2 Delta and Omicron variants and has been effective in protecting individuals against severe COVID-19-related outcomes (11–14).

In the late August 2021, the Public Health Agency of Sweden recommended a third dose of vaccine to vulnerable groups of patients such as HD patients, and this patient population was administered a third dose of vaccine in the middle of September.

Few studies have studied both the humoral (B-cell) and cellular (T-cell) response concomitantly in HD patients after three doses of SARS‑CoV‑2 BNT162b2 mRNA vaccine. One recent study reported that the third dose of SARS‑CoV‑2 BNT162b2 mRNA vaccine generates a higher serological response than the second vaccine dose in HD patients (6). The rate of decline of the immune response after three doses of vaccine remains to be investigated.

The aim of this study was, therefore, to study the humoral and cellular response to the second and third doses of SARS‑CoV‑2 BNT162b2 mRNA vaccine in HD patients and to follow the response over time.

## Methods

### Patients

In December 2020, vaccination against COVID-19 with the SARS-CoV-2 BNT162b2 mRNA vaccine (PfizerBionTech) began, and a total of 50 HD patients were enrolled in this study. The patients received their first vaccine dose from 28 December 2020 to 22 January 2021, the second dose from 20 January 2021 to 10 March 2021, and the third dose in 14–15 September 2021. The patients were tested for their immune response 1) 7–15 weeks and 2) 6–8 months after the second vaccine dose (serological response only), and 3) 3 weeks and 4) 3 months after the third dose of vaccine. This study was approved by the ethics committee (dnr 2021-00683). All study procedures were performed according to the principles of the Declaration of Helsinki, and a written informed consent was obtained from all participants.

### Analysis of SARS-CoV-2 IgG antibodies (anti-N and anti-S)

Levels of SARS-CoV-2 IgG antibodies were analyzed at the laboratory of clinical microbiology, Uppsala University Hospital, using both SARS-CoV-2 IgG II Quant assay for quantitative determination of IgG antibodies to SARS-CoV-2 (spike receptor-binding domain/anti-S) and SARS-CoV-2 IgG for qualitative determination of IgG antibodies to SARS-CoV-2 (nucleocapsid domain/anti-N) as previously described (7). The cut-off was set to 100 AU/mL (Abbott Units/milliliter) for positive result for the quantitative method, and 1, 4 S/CO (Signal to Cut-off Value) for the qualitative method. Anti-S values of 100 AU/mL or above were positive, and values between 50 and 99 AU/mL were considered borderline. Levels of antibodies below 50 AU/mL were considered negative.

### Analysis of T-cell activation with Enzyme-Linked ImmunoSpot

The analyses of cellular response to COVID-19 vaccination (T-cell reactivity) were performed by Enzyme-Linked ImmunoSpot (ELISPOT) at ABC-labs, Solna, Sweden, as described in our previous publication (7). Briefly, peripheral blood mononuclear cells (PBMC) were purified from heparinized whole blood by centrifugation and then incubated and stimulated with COVID-19 spike protein for 16 h. The immune response and T-cell activation were then quantified by measuring the level of interferon (IFN)-γ production.

### Statistical analysis

Descriptive statistics was used to describe demographical and clinical characteristics in Table 1. Means with standard deviations and medians with interquartile range (IQR) were used to describe quantitative variables. Absolute frequencies and percentages were used for categorical variables. Differences between groups were assessed with Fischer’s exact test. Serological response (anti-S) and T-cell reactivity in the graphs were log-transformed to log10. Anti-S or T-cell reactivity below the detection levels < 50 AU/mL and < 7 units was set to half the detection level in this analysis, i.e. 25 AU/mL and 3.5 units, respectively. For T-cell reactivity, some values were zero, and these were therefore assigned as 0.1 for the log transformation. When assessing between subject differences Friedman test (data not normally distributed), *P*-values representing corrections and adjustments for multiplicity with False Discovery Rate (FDR) were used (15, 16). Statistical analyses were performed using the IBM SPSS Statistics version 28 software (IBM, Armonk, NY, USA) or GraphPad Prism 9.0.2.

**Table 1:** Demographic data and clinical characteristics of patients initially included in the study (n = 50) and those who were followed during the entire study (n = 40).

Clinical variables	7–15 weeks after dose 2 (*n* = 50)	3 months after dose 3 (*n* = 40)
Age (years)	69.4 ± 14.1 (25, 90)	70.6 ± 12.5 (42, 90)
Men/women	31/19, 62%/38%	25/15, 67%/33%
Body mass index (BMI, kg/m^2^)	26.1 ± 5.6 (13, 44)	26.3 ± 6.1 (13, 44)
Dialysis duration at study start (months)	65.1 ± 74.0 (5, 470)	69.1 ± 79.8 (5, 470)
Diabetes mellitus (DM) (*n*, %)	23 (46)	19 (48)
Nefrosclerosis (*n*, %)	15 (30)	10 (25)
Autosomal dominant polycystic disease (*n*, %)	4 (8)	3 (8)
Chronic glomerulonephritis (*n*, %)	8 (16)	7 (18)
Vasculitis/anti-GBM-nephropathy (*n*, %)	4 (8)	3 (8)
Ongoing medication with CNIs (*n*, %)	1 (2)	1 (2)
Ongoing medication with MMF (*n*, %)	1 (2)	1 (2)
Previous treatment with CNI (*n*, %)	2 (4)	1 (2)
Previous treatment with rituximab or cyclophosphamide (*n*, %)	5 (10)	4 (10)
Current steroid treatment (*n*, %)	7 (14)	7 (18)

CNI; calcineurin inhibitor, GBM; glomerular basement; MMF: mycophenolate mofetil.

## Results

Demographical and clinical characteristics of the patients are presented in Table 1. Fifty patients were initially enrolled into the study. At the time of the second blood sampling after dose two, patients have died, and two had been transplanted (*n* = 46). Three weeks after dose three, two more patients had died and one had been transplanted, and two had not received the third dose. One patient refused to take a third dose, and one was deemed medically unfit to receive the vaccination. Three months after dose three, one more patient had also been transplanted, so, in summary, 40 patients had been given three doses of vaccine and had blood samples to evaluate their immune response at all time-points. One of the deaths in this study may have been related to COVID-19.

### IgG antibodies to anti-S

IgG antibodies to anti-S were detected in 37 (74%) of 50 patients, 5 (10%) had a borderline response, and 8 (16%) were negative 7–15 weeks after dose two (AntiS1). Before being given the third dose at 6–8 months after dose two (AntiS2), IgG antibodies to anti-S were detected in 24 (52%) of 46 patients, 3 (7%) had a borderline response, and 19 (41%) were negative. Three weeks after the third dose (AntiS3), IgG antibodies to anti-S were detected in 39 (95%) of 41 patients and 2 (5%) were negative, and 3 months after the third dose (AntiS4), 38 (95%) were positive and 2 (5%) remained negative in the 40 patients who were followed during the entire study.

**Figure 1:**
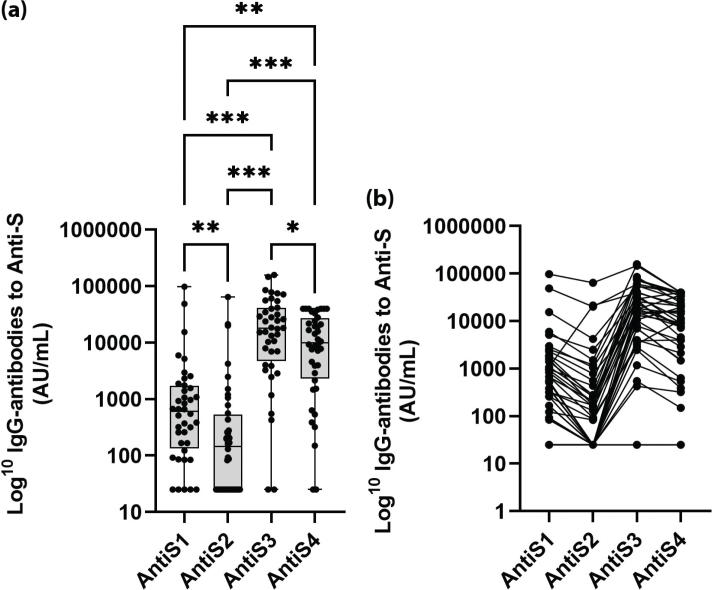
(a) IgG-antibodies against coronavirus disease 2019 (COVID-19) spike protein (Anti-S) after SARS-CoV-2 BNT162b2 mRNA vaccination 7–15 weeks (AntiS1) and 6–8 months after the second vaccine dose (AntiS2), 3 weeks (AntiS3), and 3 months after the third dose (AntiS4) in the 40 patients who remained during the entire study. (b) The individual change in IgG-antibodies against COVID-19 spike protein (Anti-S) after SARS-CoV-2 BNT162b2 mRNA vaccination in 40 patients who remained during the entire study (‘spaghetti chart’). *P < 0.05, **P < 0.01, and ***P < 0.001. P-values are adjusted for False Discovery Rate (FDR).

The anti-S response in the 40 patients who were followed during the entire study is shown in Figure 1a and b. At 7–15 weeks after dose two (AntiS1), 31 (78%) of these 40 patients were positive, 4 (10%) borderline, and 5 (12%) negative, and median anti-S titer was 606 AU/mL (IQR 134–1,712). Before being given the third dose (AntiS2), 21 (53%) were positive, 3 (7%) borderline, and 16 (40%) negative, and median anti-S titer was 146 AU/mL (IQR 25–529). Three weeks after the third dose (AntiS3), 38 (95%) were positive and 2 (5%) negative, and median anti-S titer was 17,850 AU/mL (IQR 4755–41,027). Three months after the third dose (AntiS4), 38 (95%) were positive and 2 (5%) remained negative, and median anti-S titer was 9,910 AU/mL (IQR 2,325–26,975).

A major and significant difference in anti-S response was found between the two corresponding time-points: 7–15 weeks after dose two (AntiS1) and 3 months after the third dose (AntiS4) (*P* < 0.01), as shown in Figure 1a. There was also a significant difference in the proportion of patients with a positive response at these two time points (78 vs 95%; *P* < 0.001).

### T-cell response against COVID-19 spike protein

**Figure 2:**
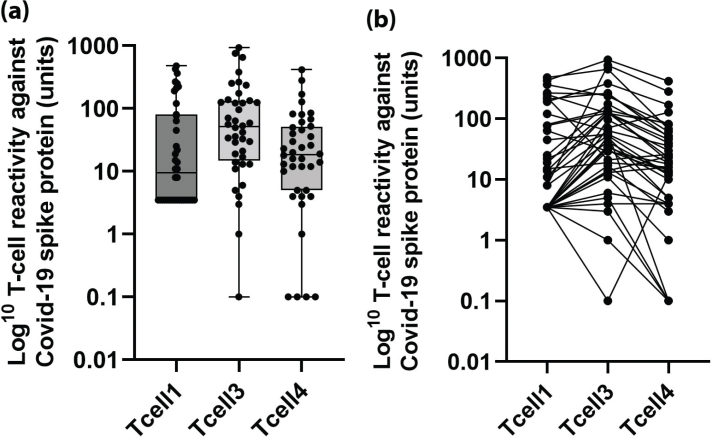
(a) T-cell reactivity against coronavirus disease 2019 (COVID-19) spike protein after SARS-CoV-2 BNT162b2 mRNA vaccine at 7–15 weeks after the second vaccine dose (Tcell1), 3 weeks (Tcell2), and 3 months after the third dose (Tcell3) in the 40 patients who remained during the entire study. (b) The individual change in T-cell reactivity against COVID-19 spike protein after SARS-CoV-2 BNT162b2 mRNA vaccine in 40 patients who remained during the entire study (‘spaghetti chart’).

A total of 29 (58%), 35 (85%), and 27 (69%) of the 50, 41, and 38 patients with valid measurements showed cellular reactivity against COVID-19 spike protein after vaccination with BNT162b2 mRNA vaccine 7–15 weeks after dose two (T-cell1) and 3 weeks (T-cell3) and 3 months (T-cell4) after the third dose, respectively. The distribution of T-cell responses against COVID-19 spike protein at the three time points for the 40 patients that remained during the entire study are displayed in Figure 2a and b. In the 40 patients who were followed during the entire study, 22 (55%), 34 (85%), and 28 (71%) patients showed cellular reactivity 7–15 weeks after second dose (T-cell1) and 3 weeks (T-cell3) and 3 months (T-cell4) after the third dose, and the median T-cell response at these time points was 9.5 (IQR 3.5–80), 51.5 (14.8–132), and 19.5 (8.8–54.2) units, respectively.

The T-cell response was numerically but not significantly higher 3 months after the third dose than 7–15 weeks (Tcell1) after the second dose (*P* = 0.96). There was a significant decline in T-cell response between 3 weeks and 3 months after the third dose (*P* < 0.001).

With regard to the two patients without an anti-S response, one patient had a weak T-cell response at 3 weeks, but at 3 months after the third dose, none of them had a T-cell response.

### IgG antibodies to anti-N and polymerase chain reaction-verified COVID-19 infections

The number of patients positive for anti-N decreased over time. Seven to 15 weeks after the second vaccine dose, seven patients (14%) were positive for anti-N, and after 6–8 months, four (9%) were positive and one (2%) had a borderline value. Three weeks and 3 months after the third dose, three patients (7%) were positive for anti-N.

Before and shortly after vaccine dose two, there were six PCR-verified COVID-19 cases in this cohort. From April 17, 2021 until January 8, 2022, no patient presented with a PCR-positive COVID-19 infection, but from January 8, six patients have tested positive for COVID-19. Two of the patients were subsequently hospitalized, but none needed intensive care. Four patients experienced only minor symptoms.

## Discussion

The main finding in this study is that a third dose of SARS‑CoV‑2 BNT162b2 mRNA vaccine resulted in a further improved both humoral and cellular immune response in HD patients than after two doses of vaccine when both IgG antibodies to anti-S and T-cell reactivity were assessed. A major and highly significant difference in anti-S response was found between 7 and 15 weeks after dose two and 3 months after the third vaccine dose.

Previous studies have shown a mixed response to two doses of SARS‑CoV‑2 BNT162b2 mRNA vaccine against COVID-19, some have found that up to 97% of the patients developed antibodies against the COVID-19 spike protein after two doses of the SARS‑CoV‑2 BNT162b2 mRNA vaccine, and others, like us, found a less pronounced humoral response of about 75–80% after two doses (7, 8). The underlying reasons for these differences are not entirely clear but could be due to different methods of measurement, different observation times, or that assessments have been done in different patient populations.

Data from Israel have shown that the effect of SARS‑CoV‑2 BNT162b2 mRNA vaccination decreases with time (9). Our data also show that the humoral response wears off with time, and after approximately 6 months after dose two of the vaccine, almost half of the patients showed a borderline or no immune response to COVID-19 spike protein.

After three doses of the SARS‑CoV‑2 BNT162b2 mRNA vaccine, the picture was quite different. Almost all patients had IgG antibodies against COVID-19 spike protein, and, in addition, most also had a ‘boosted’ T-cell response both 3 weeks and 3 months after the third dose. The data in this study suggest that both the humoral and cellular immune response to the third vaccine dose, thus, are more pronounced and sustained than after the second dose. The decline in T-cell activity seen in this study between 3 weeks and 3 months after the third dose is expected and only reflects the normal distribution kinetics of circulating T-cells to secondary lymphoid organs and effector organs. One must remember that only 2% of lymphocytes are found in the blood (17).

Even though this study is far too small to analyze how different clinical characteristics in the studied population would affect and attenuate the immunological vaccine response, it should be noted that both patients who did not show any anti-S response during the entire study were or had been treated with the immunosuppressive compound mycophenolate mofetil.

A strength in the present study is that it assesses both the humoral and cellular responses to the vaccine, measuring both levels of IgG antibodies against the SARS-CoV-2 spike receptor-binding domain (anti-S) and T-cell reactivity against COVID-19 spike protein.

However, this study has several limitations. The number of patients in this single-center study is small, and the power to detect associations between clinical characteristics and response to vaccination is, therefore, very limited. This study was designed after the vaccination started; thus, we do not have any data on antibody levels and T-cell reactivity before the vaccinations. Only 50 HD patients were included in the study, and the number of patients also decreased over time due to mortality and kidney transplantations. The design of the study had to follow the national vaccination guidelines, and, thus, the timing of the vaccination and blood sampling were not pre-determined but instead decided along the way. With all immunological studies, the clinical significance of the measured immune responses has to be established. When the vaccinations were given (December 2020–February/March 2021), the spread of COVID-19 was high, but then during most of the study period, the spread of COVID-19 was relatively low in Sweden. However, with the arrival of Omicron variant in late November–December 2021, the rate of infection in the general population increased again, and in some cases, but so far, no fatalities have occurred in our patient population.

In conclusion, HD patients have proven to be a vulnerable group during the COVID-19 pandemic (2). It is, therefore, necessary to take all possible measures and precautions to protect the HD patients against COVID-19 infections. Vaccination is an important intervention to stop HD patients from both contracting and spreading COVID-19. Our data show that a third dose of SARS‑CoV‑2 BNT162b2 mRNA vaccine gives a robust and improved both humoral B-cell and cellular T-cell immune response in most HD patients. However, a few patients did not develop any humoral B-cell response (anti-S) during the entire study. This study, therefore, also indicates the importance to monitor the vaccine response in HD-patients since those who do not respond could now be given monoclonal antibodies if they contract a COVID-19 infection or in the future be given new antivirals.

## Ethics approval and consent to participate

This study was approved by the Swedish Ethical Review Authority (dnr. 2021-00683). All study procedures were performed according to the principles of the Declaration of Helsinki, and a written informed consent was obtained from all participants.

## Availability of data and materials

All data in this paper can be obtained from the corresponding author by request.
